# Implication of the glutamate–cystine antiporter xCT in schizophrenia cases linked to impaired GSH synthesis

**DOI:** 10.1038/s41537-017-0035-3

**Published:** 2017-09-18

**Authors:** M. Fournier, A. Monin, C. Ferrari, P. S. Baumann, P. Conus, K. Do

**Affiliations:** 1 Department of Psychiatry, Center for Psychiatric Neuroscience, Lausanne University Hospital, Switzerland, Switzerland; 2 Department of Psychiatry, Service of general psychiatry, Lausanne University Hospital, Switzerland, Switzerland

## Abstract

xCT is the specific chain of the cystine/glutamate antiporter, which is widely reported to support anti-oxidant defenses in vivo. xCT is therefore at the crossroads between two processes that are involved in schizophrenia: oxidative stress and glutamatergic neurotransmission. But data from human studies implicating xCT in the illness and clarifying the upstream mechanisms of xCT imbalance are still scarce. Low glutathione (GSH) levels and genetic risk in *GCLC* (*Glutamate–Cysteine Ligase Catalytic subunit*), the gene of limiting synthesizing enzyme for GSH, are both associated with schizophrenia. In the present study, we aimed at determining if xCT regulation by the redox system is involved in schizophrenia pathophysiology. We assessed whether modulating *GCLC* expression impact on xCT expression and activity (i) in fibroblasts from patients and controls with different *GCLC* genotypes which are known to affect *GCLC* regulation and GSH levels; (ii) in rat brain glial cells, i.e., astrocytes and oligodendrocytes, with a knock-down of *GCLC*. Our results highlight that decreased *GCLC* expression leads to an upregulation of xCT levels in patients’ fibroblasts as well as in astrocytes. These results support the implication of xCT dysregulation in illness pathophysiology and further indicate that it can result from redox changes. Additionally, we showed that these anomalies may already take place at early stages of psychosis and be more prominent in a subgroup of patients with *GCLC* high-risk genotypes. These data add to the existing evidence identifying the inflammatory/redox systems as important targets to treat schizophrenia already at early stages.

## Schizophrenia: antioxidant deficit increases a key neurotransmitter transporter

Deficit of antioxidant synthesis in schizophrenia leads to oxidative stress and changes in neurotransmitter transporter. Led by Kim Do, a team of researchers from Lausanne University in Switzerland investigated the role of the cell-surface transport protein xCT in schizophrenia. They found that an enzyme responsible for antioxidant production is disturbed in patients. This leads to decreased antioxidant levels and consequently to oxidative stress—i.e. the accumulation of reactive oxygen molecules, damaging the cells component and impairing cell functioning—which in turn affects the functioning of the antioxidant pathway, including xCT. xCT, which exports the neurotransmitter glutamate, is thus overproduced in schizophrenia. The resulting increase of neurotransmitter activity, alongside the increase in oxidative stress, is thought to play a major role in the pathophysiology of schizophrenia, including at early stages of the disease.

## Introduction

The system xc- is a sodium-independent antiporter, which imports cystine and exports glutamate in a 1:1 ratio.^1^ Intracellular cystine is readily reduced to cysteine, the limiting precursor for glutathione (GSH) synthesis. Accordingly, xc- is widely reported to support anti-oxidant defenses in vivo.^2,3^ xc- is a heterodimer formed by the association of xCT (coded by *SLC7A11*) and 4F2hc (*SLC3A2*). xCT is the specific chain and an increase of gene expression often reflects an enhancement of cystine transport.^3–5^ xCT is stabilized at the membrane by CD44, a receptor for hyaluronic acid, whose expression increases intracellular levels of cysteine and GSH.^6^

Oxidative stress induces the expression of xCT.^2,3^ The transcription factor Nrf2 is well described as a master regulator for the up-regulation of genes in response to oxidative stress.^7^ In basal conditions, Keap1 binds to Nrf2 and promotes its degradation by the ubiquitin-proteasome system.^8^ In case of oxidative stress, Keap1 dissociates, allowing Nrf2 accumulation, translocation to the nucleus, and binding to antioxidant response elements (ARE) in the promoter regions of target genes.^9–11^ The promoter of *SLC7A11* contains four ARE^12,13^ and activation of *SLC7A11* expression by oxidation depends on Nrf2 as shown in Nrf2-/- mice.^12^ The Nrf2 inducer *tert*-butyl-hydroquinone (*t*BHQ), as well as the inhibitor of GSH synthesis buthionine sulfoximine (BSO), robustly increase xCT protein levels in cell culture.^14,15^

xCT expression is high in the brain^16,17^ where it is expressed by astrocytes^18^ while mature neurons show no or little expression.^2,18^ xCT in rodent brain modulates extracellular glutamate levels through non vesicular release: over half of the non synaptic release of glutamate is attributed to the antiporter.^19–21^ Changes in xCT levels are linked to many neurological and psychiatric disorders, including schizophrenia based on two human studies.^3,22,23^ In 'postmortem' tissue of dorso lateral-prefrontal cortex, xCT protein levels are increased in schizophrenia patients compared to control individuals.^24^ A recent study reported that *SLC7A11* gene expression is decreased compared to controls in peripheral white blood cells from Chinese Han patients.^25^ The authors of both studies excluded a confounding effect of antipsychotic treatment but did not identify potential upstream pathways which may lead to xCT impairment in patients.

Mounting evidence suggests oxidative stress and impairment of fast-spiking GABAergic interneurons as interdependent mechanisms forming a hub in schizophrenia physiopathology on which genetic and environmental factors converge.^26–28^ Many studies revealed markers of oxidative stress in patients, both in the brain and in peripheral samples such as blood or fibroblasts.^29^ In line with these observations, levels of the anti-oxidant defenses differ between patients and control individuals.^30^ These data indicate that induction of the response to oxidative stress, despite being present to some extent, is not efficiently regulated in patients. The gene coding for the limiting enzyme for GSH synthesis (*GCLC*; *Glutamate-Cysteine Ligase Catalytic subunit*) was associated with schizophrenia and variants of the tri-nucleotide repeat polymorphism in *GCLC* were more frequent in schizophrenia patients than in controls (*GCLC* high-risk genotypes).^31^ The *GCLC* high-risk genotypes are associated with a decrease of GSH levels in medial prefrontal cortex^32^ and in fibroblasts.^31^ Moreover a metabolomic study with patients’ fibroblasts showed altered reactivity to oxidative stress in *GCLC* high-risk genotypes.^33^

Therefore xCT is at the crossroads between oxidative stress and glutamatergic neurotransmission, two processes that are involved in schizophrenia. But data from human studies implicating xCT in the illness are still scarce, and the upstream mechanisms leading to xCT imbalance deserve further clarification. We hypothesize that oxidative stress induces xCT function, which subsequently participates in the dysregulation of glutamatergic signaling. In the present study, we aimed at determining if xCT regulation by the redox system is involved in schizophrenia physiopathology. First, we used fibroblasts from patients and controls to assess the impact of *GCLC* high-risk genotypes on xCT expression and activity, either in control conditions or by *t*BHQ-induced anti-oxidant response. Second, we investigated whether impairment of *GCLC* expression may alter xCT function in rat brain glial cells, astrocytes and oligodendrocytes.

## Results

### *GCLC* high-risk genotypes are associated with increased levels of xCT mRNA.

Fibroblasts from schizophrenia patients or control individuals, with *GCLC* high-risk or *GCLC* low-risk genotypes (Table 1), were treated or not by *t*BHQ. Gene expression was assessed by microarray in vehicle and in *t*BHQ-treated conditions.

**Table 1:** Demographics for the microarray study

	Patients		Controls	
*GCLC*	Low-risk	High-risk	Low-risk	High-risk
*n* =	10	10	15	5
Age in years (s.d)	30.3 (2.2)	36.7 (4)	38.9 (2.3)	28.8 (2.7)
Sex (males/females)	6 / 4	7 / 3	9 / 6	2 / 3
Diagnostic				
*Schizophrenia*	9	6	–	–
*Schizo-affective*	–	2	–	–
*Schizotype*	1	1	–	–
Medication in CPZ	80 (20)	79 (44)	–	–

*s.d.* standard deviation

**Fig. 1:**
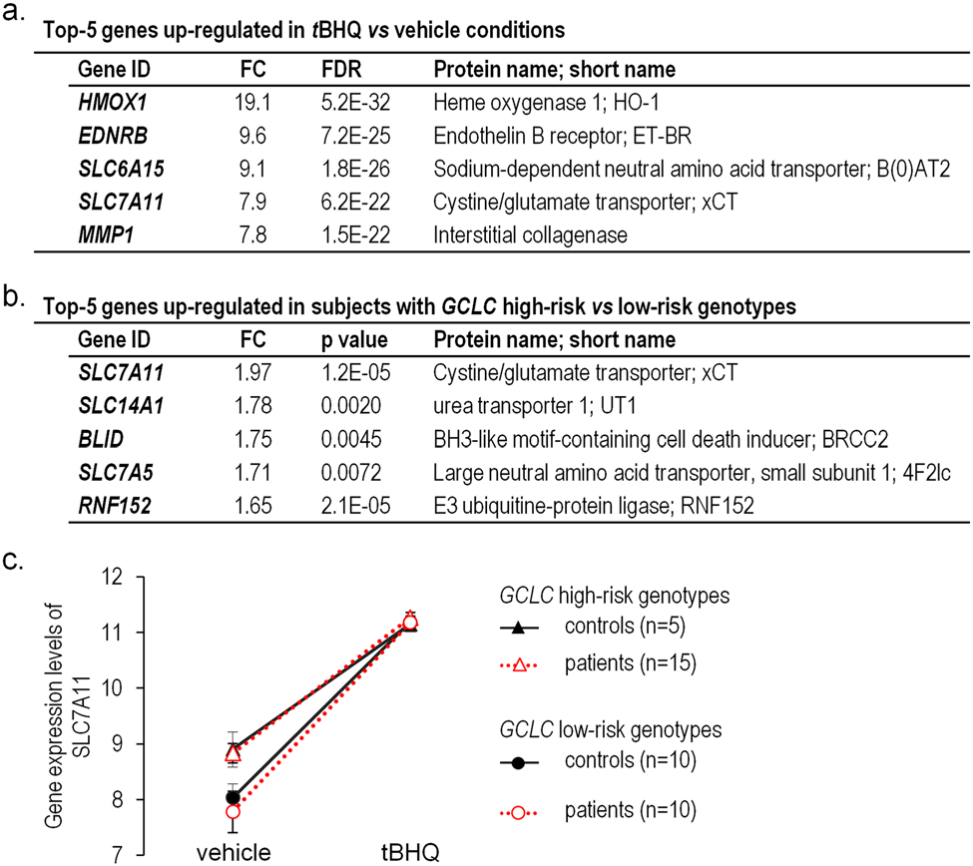
*SLC7A11* expression in skin fibroblasts with *GCLC* high-risk or *GCLC* low-risk genotypes: Top-5 genes up-regulated in fibroblasts treated by *t*BHQ versus vehicle (**a**), and up-regulated in fibroblasts with GCLC high-risk versus GCLC low-risk genotypes (**b**) both in patients and controls. *FC* fold of change, *FDR* false discovery rate (paired comparisons). **c** Plot illustrating microarray data for *SLC7A11*. Data are represented as mean ± standard error of the mean

In the top five genes that were up-regulated by *t*BHQ both in patients and controls, *SLC7A11* (coding for xCT) expression showed a 7.9-fold increase (false discovery rate for paired comparisons, FDR < 1.10^−21^, Fig. 1a). Expression of the gene *SLC3A2* (coding for 4F2hc) was also increased by *t*BHQ, suggesting an overall enhancement of amino-acid uptake (fold change, FC = 3.10; FDR = 1.10^−20^).

Levels of *SLC7A11* and *SLC3A2* were similar between patients and controls both in vehicle (FC = −1.03; *p*-value = 0.85; FC = 1.04; *p*-value = 0.73) and *t*BHQ-treated condition (FC = 1.06; *p*-value = 0.72, FC = 1.04; *p*-value = 0.70).

When comparing individuals (patients and controls) with *GCLC* high-risk and *GCLC* low-risk genotypes, *SLC7A11* was the most up-regulated gene associated with *GCLC* high-risk variants, with a 2-fold increase of expression already at basal level (*p*-value = 1.2,10^−5^, Fig. 1b). Expression of the subunit *SLC3A2* was not modified (FC = 1.22; *p*-value = 0.08), however the gene *CD44* was slightly increased (FC = 1.16; *p*-value = 0.002). For *SLC7A11*, the interaction between *t*BHQ treatment and genotype was significant (*t* = -3.81; *p*-value = 0.0004); examining the means of expression indicates that the up-regulation in response to *t*BHQ was less in the *GCLC* high-risk genotypes than in the *GCLC* low-risk genotypes (Fig. 1c).

Altogether these data indicated that regulation of xCT expression was altered in individuals with *GCLC* high-risk genotypes. Therefore *GCLC* high-risk schizophrenia patients may represent a distinct subgroup with more pronounced anomalies of xCT regulation than *GCLC* low-risk patients. In a next step, we aimed at validating this finding at the functional level in early psychosis patients. In order to maximize the power and to avoid bias due to sex, we analyzed only male early psychosis patients.

### *GCLC* high-risk genotypes are associated with increased cystine uptake

We quantified cystine uptake by xc- system in fibroblasts from *GCLC* low-risk, high-risk early psychosis patients and age-matched *GCLC* low-risk controls (Table 2), in vehicle and *t*BHQ treated conditions.

**Table 2:** Demographics for the uptake experiments

	Patients		Controls
*GCLC*	Low-risk	High-risk	Low-risk
*n *=	11	8	9
Age in years (s.d)	22.6 (3.5)	22.3 (3.2)	23.5 (3.6)
Sex (males/females)	11 / 0	8 / 0	9 / 0
Diagnostic			
*Schizophrenia*	9	6	–
*Schizo-affective*	1	1	–
*Schizophreniform*	1	1	–
Illness duration in years (sd)	3.8 (3.2)	2.0 (1.2)	–
Medication in CPZ	303 (247)	301 (265)	–

*s.d.* standard deviation, *CPZ* chlorpromazine equivalents

**Fig. 2:**
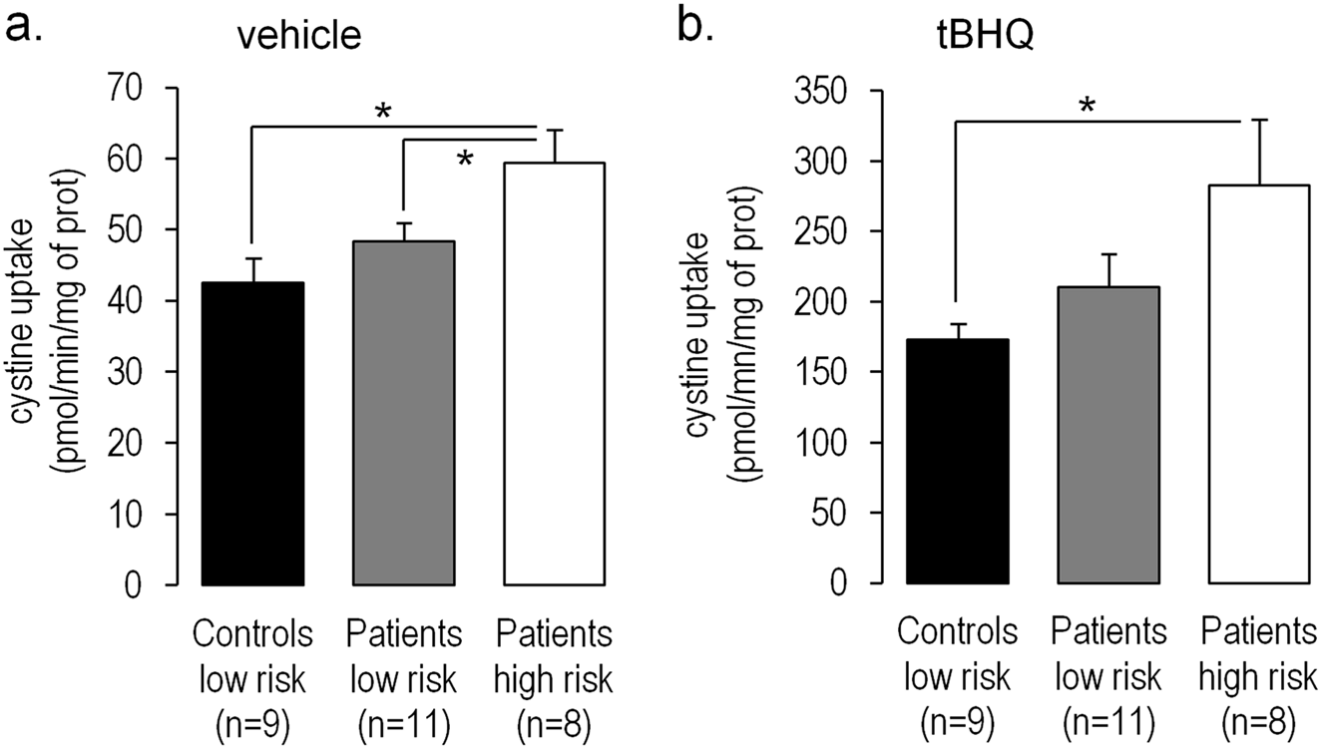
Cystine uptake by skin fibroblasts: Cells were treated (**a**) with vehicle (0.05% DMSO) or (**b**) with *t*BHQ (50uM for 18 h) which induces the anti-oxidant response. Internalized Cystine was assessed after 5 min of uptake. Data are represented as mean ± standard error of the mean; *p-value < 0.05

In vehicle conditions, cystine uptake was higher in *GCLC* high-risk patients than in *GCLC* low-risk patients and *GCLC* low-risk controls (respectively 1.4-fold; *p* = 0.010 and 1.2-fold; *p* = 0.041, see Fig. 2a). The uptake was inhibited by the addition of glutamate or by the xCT inhibitor sulfasalazine, therefore indicating the specificity of the measurements (Supplementary Fig. 1). As expected, treatment with *t*BHQ increased cystine uptake by 4-fold (*p* < 0.01). The tBHQ-induced cystine uptake was comparable for the three groups. After *t*BHQ treatment, cystine uptake remained higher in *GCLC* high-risk patients compared with *GCLC* low-risk controls (1.6-fold; *p* = 0.040, Fig. 2b), but not compared with *GCLC* low-risk patients. Cystine uptake was not correlated with the levels of anti-psychotic treatment (Supplementary Fig. 2).

Because regulation of xc- system may differ according to cell types, we wanted to clarify whether these impairments are relevant for specific brain cells.

### *GCLC* down-regulation increases cystine uptake by astrocytes

Because xc- system is mostly present in astrocytes and not in neurons,^18^ we assessed cystine uptake in glial cells from rat cortex (oligodendrocyte progenitor cells (OPCs) and astrocytes). To impair the regulation of *GCLC*, cells were transduced by lentivirus to overexpress shRNA as previously described.^34^

**Fig. 3:**
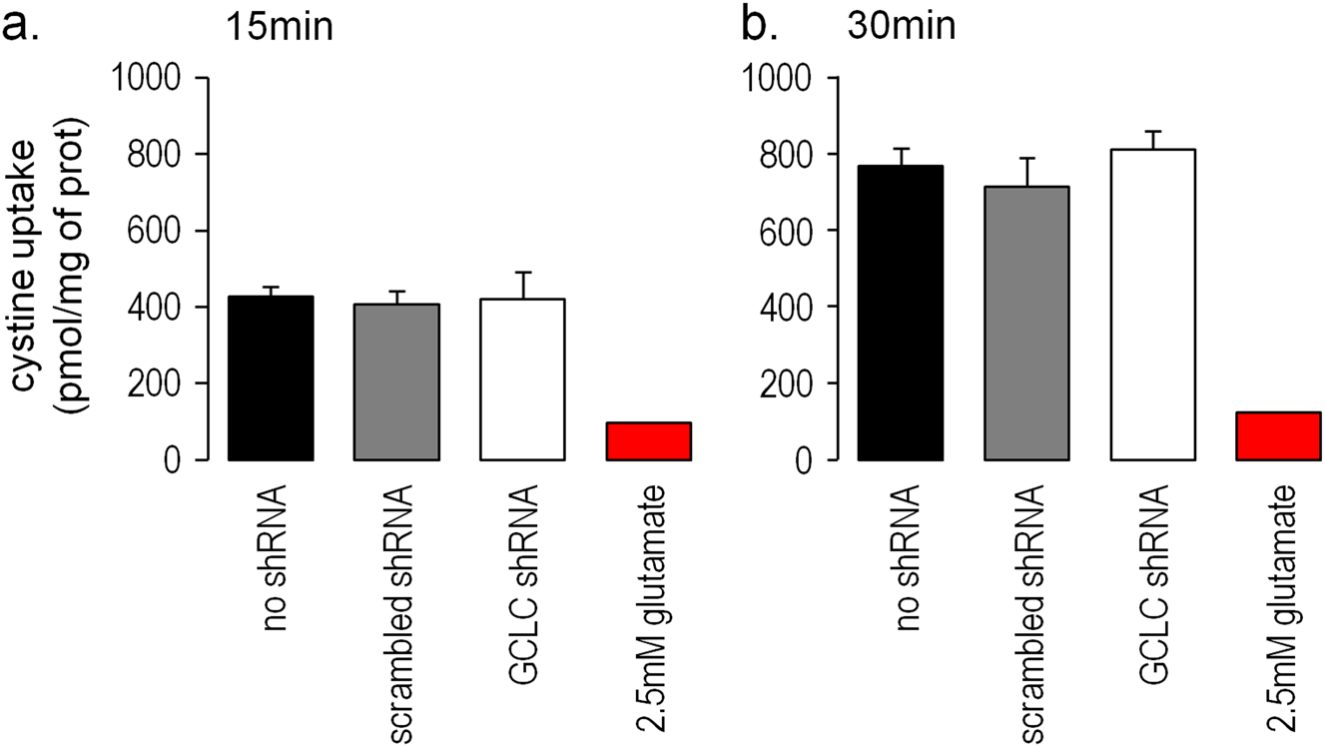
Uptake of cystine by OPCs was evaluated after 15 min (**a**) and 30 min (**b**). Three conditions were compared: without shRNA (black), transduced with a scrambled shRNA (gray), transduced with *GCLC* knock-down (white). Background level of cystine uptake was evaluated in the presence of glutamate (red). Data are represented as mean ± standard error of the mean (*n* = 4 per group); *p-value < 0.05

Knock-down with shRNA decreased GCLC protein levels by 49% in OPCs.^34^ OPCs had a slow uptake of cystine, which reached a maximum after 30 min and was inhibited by the addition of glutamate (Fig. 3). The knock-down of *GCLC* did not affect the level of cystine uptake after 15 min (Fig. 3a) nor after 30 min compared to either scrambled shRNA or non infected cells (Fig. 3b).

**Fig. 4:**
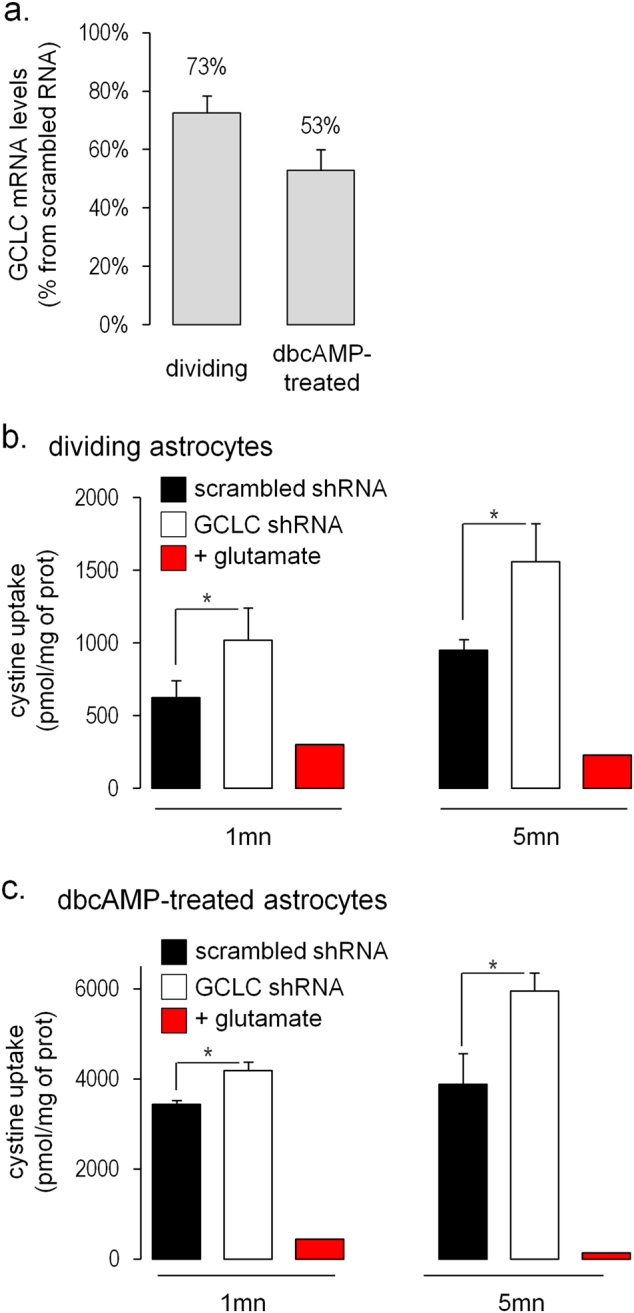
Uptake of cystine by astrocytes: **a** Decrease of *GCLC* mRNA assessed by quantitative PCR in astrocytes transduced with *GCLC* shRNA is expressed as percentage of the condition with scrambled shRNA. **b** Uptake of cystine in dividing astrocytes transduced with scrambled (black) or *GCLC* shRNA (white). **c** Uptake of cystine in dbcAMP-treated astrocytes transduced with scrambled (black) or *GCLC* shRNA (white). Data are represented as mean ± standard error of the mean (*n* = 3 per group); *p-value < 0.05

In dividing astrocytes, knock-down with shRNA decreased *GCLC* mRNA by 27% (Fig. 4a). Cystine uptake was faster in astrocytes than in OPCs, and was also inhibited by sulfasalazine (Fig. 4b). After *GCLC* knock-down, the uptake was 1.6-fold higher than in control conditions with scrambled shRNA, both after 1 and 5 min of uptake (Fig. 4b).

Primary astrocytes were also cultured with di-butyryl cyclic AMP (dbcAMP) as dbcAMP-treated astrocytes may resemble more closely the differentiated astrocytes present in brain tissue.^35^ Cyclic AMP induces morphological changes, stops cell division and increases antioxidant defenses.^35–37^ dbcAMP-treated astrocytes had a 6-fold higher uptake of cystine than dividing astrocytes (see uptake at 5 min in Fig. 4b, c and consistent with previous publications^15,38^). In dbcAMP-treated astrocytes, knock-down with shRNA decreased *GCLC* mRNA by 46% (Fig. 4a). *GCLC* knock-down in dbcAMP-treated astrocytes led to a 1.2-fold and 1.5-fold increase of cystine uptake after 1 and 5 min respectively compared with scrambled shRNA (*p* = 0.028 and 0.045 respectively; Fig. 4c).

## Discussion

We studied the regulation of xCT by genetic impairment of GSH synthesis. We found that fibroblasts with *GCLC* high-risk genotypes, which are associated with lower brain GSH brain levels and higher risk for schizophrenia^31,32^ displayed higher expression and higher activity of xCT than fibroblasts with *GCLC* low-risk genotypes. The *t*BHQ-induced increase of cystine uptake appeared to be similar across genotypes. In a translational approach, we confirmed that *GCLC* knock-down increased the activity of xCT in primary culture of rodent astrocytes. We did not observe the same response to *GCLC* knock-down in oligodendrocyte precursors, therefore underlining that xCT regulation by *GCLC* levels was cell type dependent. Altogether, the results indicate that impaired GSH synthesis leads to the upregulation of xCT activity in specific glial cells, a mechanism already relevant to early stages of schizophrenia.

These data add to the characterization of *GCLC* high-risk genotypes. Previous works indicate that these high-risk genotypes, without an additional oxidative stress, affect at least two metabolic pathways in cultured fibroblasts: the redox system^31,33^ and lysolipids levels.^33^ Importantly, the redox pathway is also affected in blood^39^ and in the brain as shown by the 14% decrease of GSH concentration in prefrontal cortex of *GCLC* high-risk individuals.^32^ Here we show that the *GCLC* high-risk genotypes are also associated with an increased activity of xCT in fibroblasts. Frequencies of *GCLC* high-risk genotypes are higher in schizophrenia patients than in controls^31^ and vary with the ethnicity.^40^ Controlling for this confounding factor in case-control studies is thus important as it may explain discrepancies between studies.

The decrease of GCLC expression (by genetic variants or by knock-down) may increase xc- activity through the Nrf2 signaling pathway. Indeed *GCLC* expression tightly controls GSH levels, and the high-risk variants are associated with lower GSH.^31,32^ Depletion of cysteine or GSH may lead to oxidative stress, to the activation of Nrf2, and to the enhancement of xCT activity.^12^ Consistently, previous work showed that depletion of intracellular cysteine or GSH enhanced the activity of the cystine-glutamate antiporter.^15,41^ Although Nrf2 is the most studied antioxidant regulator, other pathways may also be involved.^15^ For instance, *SLC7A11* promoter also contains binding sites for ATF4, a transcription factor typically activated by amino acid starvation.^42^ Activation of ATF4 pathway up-regulates xCT, increases intracellular GSH levels, and confers resistance to oxidative stress.^43^

An interesting downstream effect of enhanced xc- activity is the increased efflux of glutamate, which may participate to schizophrenia physiopathology by affecting the inhibitory/excitatory balance.^22^ Glutamate levels in various brain regions are higher in early psychosis patients than in matched healthy controls.^44^ Accordingly, impairment of glutamate transport has been suggested by 'postmortem' brain studies of schizophrenia patients.^45,46^ The upregulation of xCT may thus participate in the impairment of glutamate transport and in the increase of brain glutamate in schizophrenia. xc- activity has been shown to significantly affect glutamate levels as knock-out mice for *Slc7a11* have decreased levels of extracellular glutamate.^20,21^ Gene deletion leads to minor spatial memory deficits,^20,47^ and impaired hippocampal LTP^47^ and acute inhibition of xc- is associated with anxiety-related behaviors.^49^ Inactivation of the glial glutamate transporter GLAST, which likely leads to an increase of extracellular glutamate, also leads to endophenotypes that are associated with schizophrenia such as memory deficits and impaired acoustic startle.^47^

Interpretation of the results is limited by the use of rodent cells, as it is not clear to which extent rat primary glial cells reflect human brain physiology. Because xCT is induced by the higher O_2_ levels in culture compared to in vivo conditions,^3^ interpretation of this work is limited by the in vitro setting. Nevertheless, regulatory mechanisms of xCT expression have been largely deciphered by studies using cell culture,^1^ thus highlighting that our conclusions can be transposed to in vivo conditions. Moreover, our observation that OPCs did not display an increase of xCT function following *GCLC* knock-down is also in favor of a specific regulation.

In conclusion, our data show that a decrease of *GCLC* expression, the limiting synthesizing enzyme for GSH, leads to an upregulation of xCT levels in patients’ fibroblasts as well as in astrocytes. These results from schizophrenia patients support the emerging data involving xCT dysregulation in illness physiopathology and further indicate that it can result from redox changes such as lower GSH levels, which have been previously associated with schizophrenia. According to our results, xCT dysregulation already takes place at early stages of psychosis and is more prominent in a subgroup of patients with *GCLC* high-risk genotypes. Investigating consequences of xCT dysregulation at the clinical level would shed light on the symptoms that may respond to molecules targeting the immune and/or redox system.

## Methods

### Recruitment

All individuals were recruited in Lausanne area, Switzerland.^31,33^ Early psychosis patients were diagnosed according to DSM-IV criteria after a 3-years follow-up in the TIPP program (University Hospital Lausanne^33,50^). Patients included in the TIPP program had less than 6 months of anti-psychotic medication. Less female than male patients have been recruited in this cohort; therefore we focused on men the study of early psychosis patients. Control subjects were assessed and selected with the Diagnostic Interview for Genetic Studies. Individuals with a neurological, major mood, psychotic, or substance-use disorder and a first-degree relative with a psychotic disorder were excluded. All enrolled subjects provided a fully informed written consent; all procedures, including biopsy, were in accordance with the ethical standards of the Helsinki Declaration as revised in 1983, and was approved by the ethical committee of Lausanne University Hospital on human experimentation.

### Culture of fibroblast

Secondary cultures of fibroblasts were established from skin biopsies.^31,33^ Skin-derived fibroblasts from patients with early psychosis and age-matched, sex-matched controls were processed in parallel as described previously.^31,33^ We could not match for *GCLC* genotypes as control individuals with *GCLC* high-risk genotypes were not frequent enough in our cohort. After thawing, cells were amplified in Dulbecco’s Modified Eagle Medium (DMEM, Gibco), 2% Ultroser™G (Pall Corp), 1% penicilline-streptomycine (Gibco). After five passages, we plated the fibroblasts at 5,10^4^ cells/well (12-wells plate); we treated the cells the day after (18 h of treatment, 50uM *t*BHQ (Sigma) or 0.05% dimethylsulfoxide (Sigma) for vehicle).

### Microarray

RNA was purified with RNAeasy column (Qiagen); quality was checked by Aligent 2100 bioanalyzer chips. Affymetrix, 1.0ST GeneChips were processed at Lausanne Genomic Technologies Facility according to manufacturer recommendation. Hybridization quality was assessed using the Bioconductor package affy^51^ in R (http://www.R-project.org; http://www.Bioconductor.org). Log2 normalized expression signals were calculated using RMA algorithm^52^ (comprising background correction, quantile normalization and probe set summary by robust regression). Sub-sequent analyses were based on Gene Ontology Annotation (UniProt-GOA).

### Primary cultures of glial cells

All animal procedures were approved by the Swiss cantonal veterinary office. Primary glial cells were dissociated from cortex of males and females Wistar Han rat pups at 3-days postnatal as previously described.^34^ Cells were cultured in DMEM, 10% fetal calf serum (FCS), 1% penicillin-streptomycin at 37 °C, 5% CO_2_. After 7 days in vitro (DIV), cells were infected with lentiviruses (multiplicity of infection: 5) to overexpress *GCLC* shRNA: GGAGGCTACTTCTATATTA or scrambled shRNA: CTTACAATCAGACTGGCGA. Puromycin was added 48 h post-infection (Calbiochem, 1ug/mL). After 10 DIV, oligodendrocytes progenitor cells (OPCs) were separated from astrocytes and microglia by overnight shaking. OPCs were plated at 1.2,10^5^ cells/well in 12-wells plate (DMEM, 2.5 uM forskoline (Calbiochem), 50 ug/ml apotransferrin, 5 ug/ml insulin (Sigma), 30 nM sodium selenate (Sigma), 10 nM hydrocortisone (Sigma), 10 nM D-biotine (Sigma), 1 mg/ml bovine serum albumin (Sigma), 10 ng/ml PDGF-AA (Sigma), 10ng/ml human fibroblast growth factor-basic (Sigma)); experiments were performed 14 DIV. In parallel, astrocytes which remained attached after shaking were plated at 7.5,10^4^ cells/well in 12-wells plates in normal culture media (DMEM, 10% FCS, 1% penicillin-streptomycin) or in differentiation media (DMEM, 2% FCS, 15uM di-butyryl cyclic AMP (Enzo) for 8 days).

### Cystine uptake

Xc- activity was assessed based on previously published protocol.^53^ Briefly, cells were washed with Hank’s balanced salt solution pH 7.4 (HBSS; 120 mM NaCl, 5.4 mM KCl, 0.8 mM MgCl_2_, 1.8 mM CaCl_2_, 0.1% Glucose, 20 mM Hepes (Sigma)) and equilibrated in HBSS for 10 min at 37 °C, eventually with transporter inhibitor (sulfasalazine 1 mM (Sigma); glutamate 2.5 mM). Medium was then changed for uptake buffer (0.5 mM acivicin, 1 mM D-aspartate, 35uM cystine including 1uCi/mL of ^14^C-cystine (PerkinElmer) in HBSS). Uptake was done at 37 °C and terminated on ice by removing uptake buffer and adding cold phosphate buffer saline (PBS). Cells were lysed with 500 μL of warm PBS with 0.5% sodium-dodecyl-sulfate. Incorporated radioactivity was quantified by liquid scintillation counting (Tricarb 2900TR Packard). Levels of radioactivity are normalized for protein content assessed with Bicinchoninic acid assay, the mean of technical duplicates was calculated. Data presented are representative of two experimental replications

### Statistical analyses

For microarray data, we calculated for each probe set *M*-values (log base 2 of the fold change between two conditions), moderated t-statistic (ratio of the *M*-value to its standard error), *p*-values derived from moderated *t*, and adjusted *p*-value (FDR, Benjamini–Hochberg step-up procedure). For uptake experiments, we used student *t*-test in R on log-transformed data.

### Data availability statement

The datasets generated during and/or analyzed during the current study are available from the corresponding author on reasonable request.

## Electronic supplementary material

Supplementary Figure 1

Supplementary Figure 2
