# Supplementary figures and images for: Implication of the glutamate–cystine antiporter xCT in schizophrenia cases linked to impaired GSH synthesis
Source: NPJ Schizophr. 2017 Sep 18;3:31. doi: 10.1038/s41537-017-0035-3 (PMC5603608; doi:10.1038/s41537-017-0035-3)

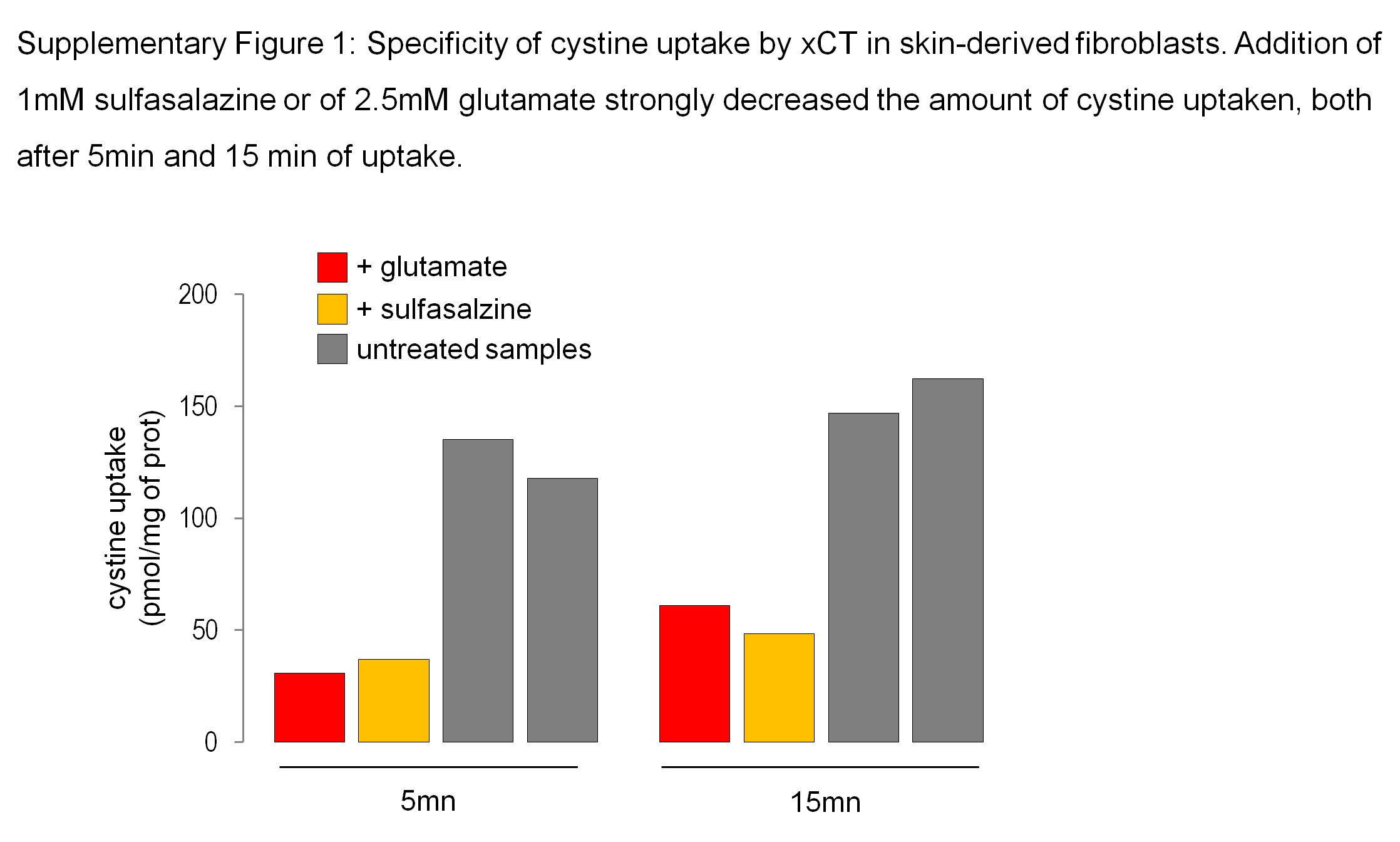

Supplement: Supplementary file 1 — Supplementary Figure 1 [file 41537_2017_35_MOESM1_ESM.tif]

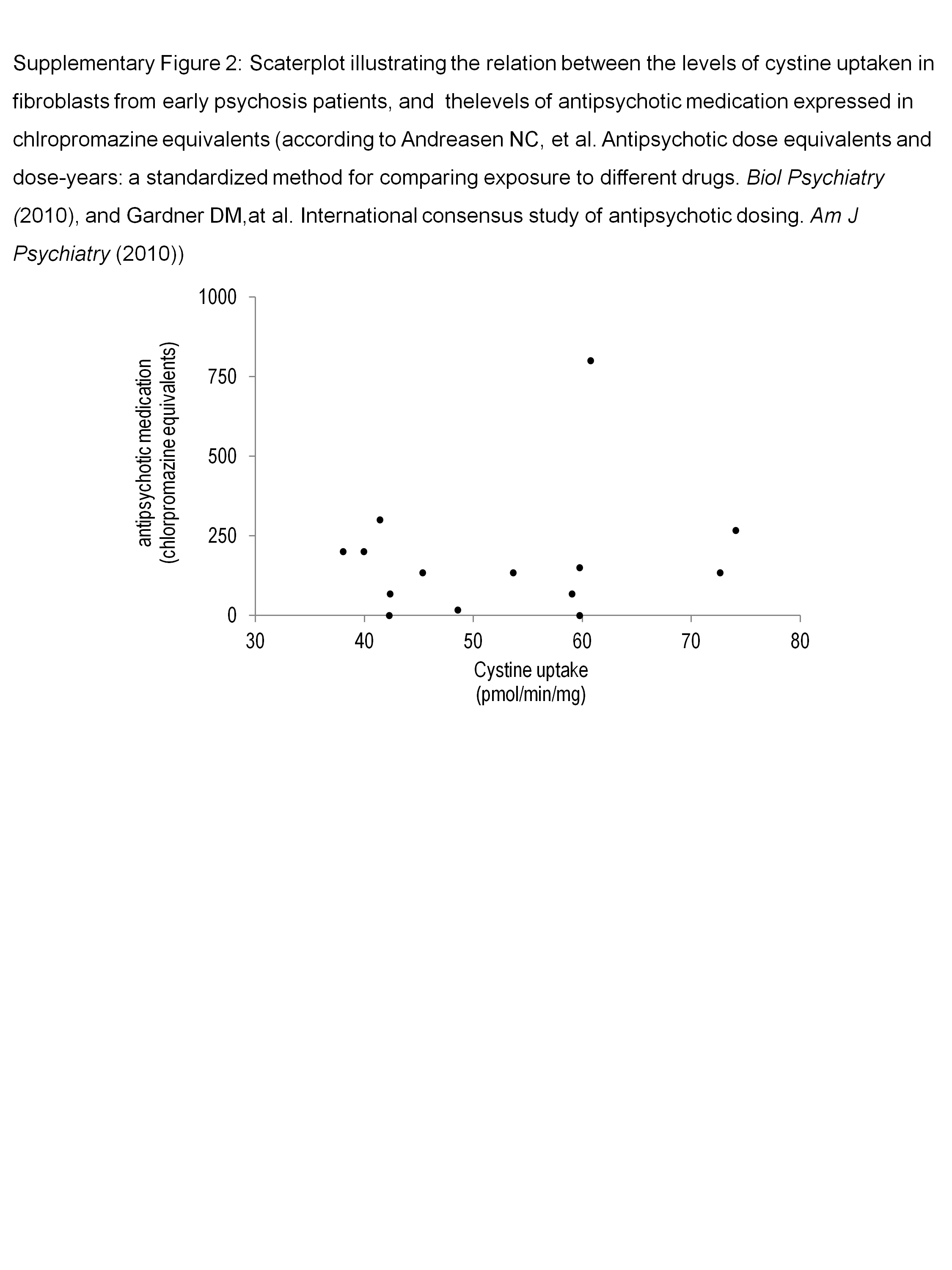

Supplement: Supplementary file 2 — Supplementary Figure 2 [file 41537_2017_35_MOESM2_ESM.tif]
